# Anisakicidal Effects of R (+) Limonene: An Alternative to Freezing Treatment in the Industrial Anchovy Marinating Process

**DOI:** 10.3390/foods11081121

**Published:** 2022-04-13

**Authors:** Luca Nalbone, Felice Panebianco, Gaetano Cammilleri, Vincenzo Ferrantelli, Filippo Giarratana

**Affiliations:** 1 Department of Veterinary Sciences, University of Messina, Polo Universitario dell’Annunziata, 98168 Messina, Italy; lnalbone@unime.it; 2 Department of Veterinary Sciences, University of Turin, Largo Braccini 2, Grugliasco, 10095 Turin, Italy; felice.panebianco@unito.it; 3 Istituto Zooprofilattico Sperimentale della Sicilia, via Gino Marinuzzi 3, 90129 Palermo, Italy; gaetano.cammilleri86@gmail.com (G.C.); vincenzo.ferrantelli@izssicilia.it (V.F.)

**Keywords:** *Anisakis*, limonene, marinated fishery products, nematicidal activity, anisakicidal activity, sensory influence

## Abstract

Anisakiasis is a fish-borne zoonotic disease caused by the ingestion of raw/undercooked fishes or cephalopods parasitized by members of the genus *Anisakis*. Freezing ensures the inactivation of viable *Anisakis* larvae; however, since it affects the organoleptic properties of food, essential oils and their compounds were proposed as an alternative. In this study, fresh anchovy fillets were experimentally parasitized with L3 *Anisakis* larvae to test the anisakicidal efficacy of R (+) limonene (LMN) in marinated fishery products. The anisakicidal effectiveness and organoleptic influence of several LMN concentrations (0.5%, 1%, and 5%) were tested during the marinating process (MS) and storage in sunflower seed oil (SO) of marinated anchovy fillets. Double treatment (DT) with 1% LMN was also performed both during marination and subsequent storage in oil. MS treatment resulted only in a reduction in larvae viability after 48 h, while a complete inactivation was observed in SO after 8, 10, and 20 days of treatment with 5%, 1%, and 0.5% LMN, respectively. DT was the most effective with complete larval inactivation after 7 days. Only 5% LMN influenced the sensory characteristics of the fillets, resulting, however, in a pleasant lemon-like odor and taste. Considering the results obtained, LMN might be a suitable natural alternative to manage *Anisakis* risk in the fishery industry.

## 1. Introduction

Anisakidosis (anisakiasis if caused by *Anisakis* spp.) is a fish-borne zoonotic disease caused by members of the family Anisakidae (genera *Anisakis*, *Pseudoterranova* and, rarely, *Contracaecum*), and species of the genus *Anisakis* (Dujardin, 1845) are considered to be mainly responsible for this zoonosis [1]. In fact, 97% of the reported cases of human anisakidosis are related to third-stage (L3) larvae of the species *Anisakis simplex sensu stricto* and *Anisakis pegreffii* [1,2].

The parasite’s life cycle involves marine mammals as final hosts, planktonic and benthic crustaceans as the first intermediate/paratenic hosts, and fishes and cephalopods as the second intermediate/paratenic hosts [3]. *Anisakis* is found worldwide and has been isolated from more than 200 fishes and 25 cephalopods [4,5,6]. In European waters, the most common hosts are the silver scabbard fish (*Lepidopus caudatus*), European hake (*Merluccius merluccius*), European anchovy (*Engraulis encrasicolus*), European pilchard (*Sardina pilchardus*), horse mackerel (*Trachurus trachurus*), and Atlantic mackerel (*Scomber scombrus*). Instead, herring (*Clupea harengus*), saithe (*Pollachius virens*), cod (*Gadus morhua*), redfish (*Sebastes marinus*), and European sprat (*Sprattus sprattus*) are reported as the commonest hosts in the Atlantic and the North Sea [2,6].

In the second intermediate/paratenic hosts, once ingested, the L3 larvae pass through the gastrointestinal wall into the celomatic cavity and encapsulate in the viscera and, less frequently, in the musculature [1,7]. Humans represent an accidental host and acquire the infection by eating fish or squid parasitized by the L3 larvae, consumed raw (e.g., sushi, sashimi, and tartare), marinated (e.g., ceviche, marinated anchovy, and gravlax), inadequately cooked, or undercooked [8,9,10]. Once ingested, live larvae try to attach to the gastric or the intestinal mucosa. The different types of anisakidosis are related to the localizations (gastric, intestinal, and extraintestinal—“visceral *larva migrans* syndrome”) and symptoms (gastrointestinal, allergic, and gastroallergic) [8,10,11]. All the reported types of anisakidosis are consequences of the ingestion of live larvae, while the allergic one (Type I immune hypersensitivity) is due to larval allergens present in the live and dead parasites [12,13,14]. Dermatitis, conjunctivitis, and asthma, not related to the ingestion, but to direct contact with the Anisakidae larvae, are reported as “occupational allergies” in fishermen, fishmongers, and fish-industry workers [15,16].

To date, the real diffusion of anisakidosis in humans is unknown due to the lack of epidemiological surveillance, and the cases are likely to be underestimated [17,18]. According to the last report of the European Food Safety Authority (EFSA) Panel on Biological Hazards (BIOHAZ) [10], a total of 20,000 anisakidosis cases were reported before 2010 worldwide, and over 90% were reported in Japan with most of the rest from European countries, such as Spain, Italy, and, less frequently, the Netherlands and Germany. In Europe, a total of 236 cases of anisakidosis were reported between 2000 and 2017, and the highest incidence was reported in Spain, where there is a greater consumption of marinated anchovies, which are traditionally prepared in vinegar in a typical dish called “bequerones en vinaigre” [19,20]. Also in Italy, marinated anchovies, called “alici marinate”, are mainly responsible for this zoonosis [9,21,22]. Guardone et al. [23] have observed that most cases of human anisakidosis in Italy are caused by L3 larvae of *A. pegreffii,* a species mostly reported in marinated anchovies.

The marinating process (with vinegar, lemon juice, or brine) is not effective in *Anisakis* devitalization [10,24]; therefore, a preventive freezing treatment (−20 °C for at least 24 h or −35 °C for at least 15 h) of raw anchovies is needed to kill viable parasites. This freezing step is mandatory for industrial and restaurant preparations according to Regulation (EC) No. 1276/2011 [25] and the “Food and Drug Administration’s guidance” [26]. Therefore, the role of the marinated anchovies in the spread of anisakidosis stems from the failure of this mandatory treatment, an occurrence most frequently reported during homemade preparation and less often in mass catering [8,9,10,18,22,27].

Since freezing and thawing affects the organoleptic properties of marinated anchovies [28,29], several alternative procedures have been proposed to obtain an equivalent anisakicidal effect [30,31,32]. The addition of different natural substances (plant extracts, essential oils, and their compounds) characterized by strong anisakicidal activity has been proposed as an alternative to the freezing treatment in marinated seafood products [33,34,35,36,37,38]. However, the application of these natural compounds in foodstuffs is limited due to their influence on the sensory characteristics of the treated products [39,40,41,42]. Among the tested anisakicidal substances, R (+) limonene (LMN), due to its safe status, pleasant smell, and lemon-like taste, represents a natural compound compatible with marinated fishery products, considering that the marinade can also be made with lemon as an alternative or in association with vinegar [34,43].

The present study aimed to evaluate the addition of R (+) LMN during the industrial anchovy marinating process as an alternative to the freezing treatment for *Anisakis* risk management.

## 2. Materials and Methods

### 2.1. Experimental Plan and Media Preparation

**Figure 1:**
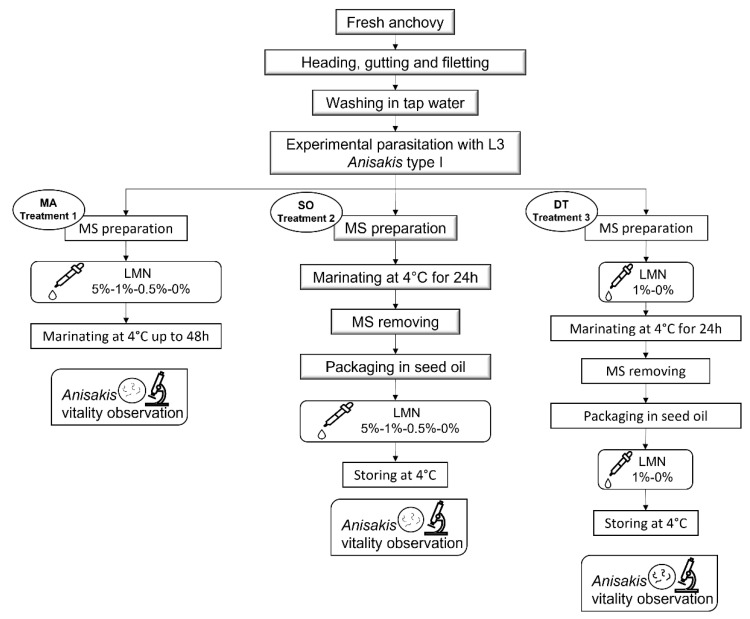
Flow chart of the different treatments with R (+) limonene (LMN) in the traditional industrial marinating process of anchovy fillets. MA: marinating; MS: marinating solution; SO: sunflower seed oil; DT: double treatment.

Based on the in vitro results reported by Giarratana et al. [34] and considering the traditional industrial anchovy marinating process (Figure S1) [44], the anisakicidal effectiveness and the sensory influence of the addition of several LMN concentrations (0.5%, 1%, and 5%) on anchovy fillets were preliminarily tested during “Marinating at 4 °C” (MA-Treatment 1) and “Storage in sunflower seed oil at 4 °C” (SO-Treatment 2) (Figure 1). The LMN concentration with the best compromise between anisakicidal efficacy and influence on the organoleptic characteristic of anchovy fillets was used in a double treatment (DT-Treatment 3) both during “Marinating at 4 °C” and “Storage in sunflower seed oil at 4 °C” (Figure 1). R (+) LMN (C_10_H_16_, PubChem CID 440917, molecular weight 136.238 g/mol) was supplied by Sigma Aldrich (Milan, Italy), while sunflower seed oil (SSO) (Carapelli-Giglio Oro, Firenze, Italy) was purchased from a large retail chain. The marinating solution (MS) was prepared according to the formulation 1:1 (*vol*/*vol*) of tap water and wine vinegar (6% acetic acid; Ponti S.P.A., Ghemme, Italy), 30 g/L NaCl (Tesauro sale srl, Terme Vigliatore, Italy), and 10 g/L citric acid (Labochimica srl, Campodarsego, Italy), used by several Italian producers [44]. The digestion solution (DS) was prepared at 0.5% *w*/*v* of pepsin (Sigma Aldrich, Milan, Italy) in 0.063 M HCl (Sigma Aldrich, Milan, Italy), just before each test.

### 2.2. Anisakis and Anchovy Fillet Collection

Fresh European anchovies (*Engraulis encrasicolus*; Linneo, 1758) were collected from local markets (Messina, Italy) and processed within 12 h of harvesting. Each anchovy was gutted, headed, and reduced into fillets just before use.

*Anisakis* larvae were collected from several specimens of silver scabbard fish (*Lepidopus caudatus*; Euphrasen, 1788) caught in the Straits of Messina (Sicily, Italy, FAO area 37) within 6 h from each test. Silver scabbard fish was selected since it is easily available within hours of fishing and normally parasitized (up to 100%) by larvae of *A. pegreffii*, which is the species of *Anisakis* commonly detected in European anchovy [9,45,46].

Once the fish arrived in the laboratory, their celomatic cavity and inner organs were examined to detect and collect *Anisakis* larvae by using needles or micro-brushes. The collected nematodes were rinsed with sterile saline solution (NaCl 9 g/L) and then carefully observed under the stereomicroscope (Leica M 205C, Wetzlar, Germany) for their integrity and viability and to confirm that they belonged to *Anisakis* L3 type I according to the differential morphological features [1]. A total of five sessions of “*Anisakis* collection” were performed. For each session, two or three morphologically confirmed *Anisakis* L3 type I (for a total of 14) specimens were placed in individual Eppendorf tubes with 70% ethanol and sent for molecular identification at the laboratories of the “Centro Italiano di Referenza Nazionale per le Anisakiasi” of the Istituto Zooprofilattico Sperimentale della Sicilia “A. Mirri” Palermo, Italy.

#### Anisakis Molecular Identification

The larvae were examined for molecular identification by PCR-RFLP according to the protocols of Ferrantelli et al. [46]. The larvae were fragmented with a scalpel, stored at −20 °C overnight, and subjected to the extraction of DNA using commercial kits based on affinity columns (Sigma Aldrich, St. Louis, MO, USA). The DNA extracted was quantified using a Nanodrop spectrophotometer (Thermo, Waltham, MA, USA). The nuclear rDNA containing the ITS region was amplified using NC5 (5′-GTAGGTGAACCT GCGGAAGGATCATT-3′) and NC2 (5′-TTAGTTTCTTTTCCTCCGCT- 3′) primers. The PCR mix was carried out as follows: 2 mM MgCl_2_, 0,2 mM of each dNTP, 20 pmol/μL of each primer, buffer AmpliTaq Gold 1X, 3.0 U AmpliTaq Gold DNA Polymerase (AB), and 20–25 ng of genomic DNA, in a final volume of 50 μL. The thermal profile of the PCR was performed as follows: 10 min at 95 °C, 35 cycles of 30 s at 95 °C, 30 s at 58 °C, and 75 s at 72 °C, followed by a final elongation of 15 min at 72 °C on a Thermal Cycler 2720 (Applied Biosystems).

The PCR products were separated by electrophoresis in 1% agarose gel, stained with SYBR safe^®^ (Invitrogen, Carlsbad, CA, USA) in Tris–borate–EDTA buffer, and visualized using a UV transilluminator (GelDoc Imaging System, Euroclone, Pero, Italy). The PCR amplification products were subjected to RFLP using two restriction enzymes *HhaI* and *HinfI* for the identification of *Anisakis* species [47]. All restriction reactions were carried out in a final volume of 20 μL containing 3 μL of DNA, 13.8 μL of distilled water, 1 μL of restriction enzyme, 2 μL of enzyme buffer, and 0.2 μL of BSA. The digestion was performed on a Thermal Cycler 2720 (Applied Biosystems, Waltham, MA, USA) overnight at 37 °C. The digestion products were electrophoresed in 2% agarose gel (Invitrogen, Carlsbad, CA, USA) stained with SYBR Safe^®^ and visualized using a UV transilluminator.

### 2.3. Preparation of Fillets Experimentally Parasitized with Anisakis Larvae

For the 3 different treatments (MA-Treatment 1, SO-Treatment 2, and DT-Treatment 3) performed in triplicate, a total of 558 anchovy fillets were experimentally parasitized with 1116 L3 *Anisakis* larvae type I according to a modified protocol proposed by Giarratana et al. [34]. Specifically, two pockets in the thickness of the epaxial musculature on each fillet were incised (in anterior and posterior portions) to contain a larva each (two larvae on each fillet). The larvae were inserted into the obtained pocket using micro-brushes and immediately closed with a commercial solution of cyanoacrylamide (Loctite, Italy), avoiding direct contact with the larvae.

### 2.4. Evaluation of Anisakis Viability

Over the years, several protocols have been implemented to evaluate the effects of different treatments on the *Anisakis* viability [48,49,50,51,52], and they have been used to implement our new protocol.

At the end of each treatment (MA-Treatment 1, SO-Treatment 2, and DT-Treatment 3), all the *Anisakis* larvae, removed from the pockets of the fillets using micro-brushes and needles, were placed into plastic Petri dishes (90 mm diameter) containing 15 mL of saline solution (NaCl 9 g/L), for 5 min to remove the potential residue of the tested solution. For the evaluation of *Anisakis* viability, the larvae were then transferred into plastic Petri dishes (90 mm diameter) containing 15 mL of the DS and maintained at room temperature (~20 °C), since these conditions stimulate *Anisakis* motility [52,53].

According to several authors, *Anisakis* larvae are considered “viable” when physically intact (integrity of cuticle and internal organs) and motile, as demonstrated by spontaneous movements upon repeated mechanical stimulation with forceps or needles [10,48].

The larvae were video recorded for 1 h by using a stereomicroscope (Leica M 205 C) equipped with Leica Application Suite eX Version 3.0.4, to evaluate their viability using the classification of Hirasa and Takemasa [51] implemented according to Guan et al. [50] (Table 1). Guan et al., using a medium with 2.5 g/L of agar, distinguished “larval mobility”, defined as “the total distance that a larva migrated inside the medium”, from “larval motility”, which was the “in situ movements” of different parts of the larval body (head part, middle part, and tail part) [50].

Based on movements observed in the DS with time-lapse video, a “viability score” from 3 to 0 was assigned to each larva (Table 1). In order to distinguish dead larvae (score 0) from those motile only after stimulation (score 1), they were mechanically stimulated with a needle at least three times. Larvae were considered dead (score 0) when no mobility was observed under the stereomicroscope for five minutes, even after stimulation. The presence of injuries in all the dead *Anisakis* larvae was also evaluated according to the following damage classification: (i) slight damage of the cuticle; (ii) interruption of the digestive tract; (iii) continuous lesion of the cuticle; (iv) continuous lesion of the cuticle associated with a leak of the digestive tract.

### 2.5. Treatment 1: Addition of LMN during Marinating at 4 °C

To assess the anisakicidal effectiveness of R (+) LMN during MA-Treatment 1 the following LMN concentrations were tested: 5%, 1%, 0.5%, and 0% as control (CTL-MA). For each concentration, a total of 18 anchovy fillets experimentally parasitized with 36 *Anisakis* larvae (2 larvae in each fillet) were marinated in 300 mL of the MS under refrigeration, and larval viability was monitored after 8, 16, 24, 32, 40, and 48 h. Usually, the industrial marinating process lasts for 24 h, but in the present study, it was protracted up to 48 h to better evaluate larval viability (Figure 1).

This treatment was carried out three times in separate conditions and at different times (at each time point, 6 *Anisakis* larvae from 3 anchovy fillets were analyzed for each replicate) for a total of 216 anchovy fillets experimentally parasitized with 432 *Anisakis* larvae.

### 2.6. Treatment 2: Addition of LMN during Storage in Sunflower Seed Oil at 4 °C

In SO-Treatment 2, the LMN was added into SO commonly used to store the marinated anchovies. A total of 84 anchovy fillets experimentally parasitized with 168 *Anisakis* (2 larvae in each fillet) were marinated in 1200 mL of MS (without LMN) for 24 h under refrigeration. The obtained marinated fillets were divided into four different groups, placed in closed plastic boxes, and submerged into SO with 5%, 1%, 0.5%, and 0% (CTL-SO) LMN concentrations. The fillets were maintained under refrigerated conditions, and the larval viability was monitored after 2, 4, 6, 8, 10, 15, and 20 days (Figure 1).

This treatment was carried out in triplicate in separate conditions and at different times (at each time point, 6 *Anisakis* larvae from 3 anchovy fillets were analyzed for each replicate) for a total of 252 anchovy fillets experimentally parasitized with 504 *Anisakis* larvae.

### 2.7. Treatment 3: Double Treatment with LMN

In DT-Treatment 3, the addition of 1% of LMN (selected as the best compromise between anisakicidal efficacy and sensory influence) was tested both during the marinating process and in subsequent storage in sunflower seed oil. A total of 30 anchovy fillets experimentally parasitized with 60 *Anisakis* (2 larvae in each fillet) were divided into two groups and marinated respectively in 300 mL of MS with two LMN concentrations (1% and 0% as DT-CTL) for 24 h under refrigeration. Each group of marinated fillets was then placed in closed plastic boxes and submerged with sunflower seed oil with 1% and 0% (always as DT-CTL) of LMN concentration, respectively. The fillets were maintained under refrigerated conditions, and the larval viability was monitored after 4, 5, 6, 7, and 8 days (Figure 1).

This treatment was carried out in triplicate in separate conditions and at different times (at each time point, 6 *Anisakis* larvae from 3 anchovy fillets were analyzed for each replicate) on a total of 90 anchovy fillets experimentally parasitized with 180 *Anisakis* larvae.

### 2.8. Sensory Evaluation of Marinated Anchovy Fillets

For each treatment, further non-experimentally parasitized fillets were prepared and processed for sensory analysis. Anchovy fillets prepared for the sensory evaluation of MA-Treatment 1 were analyzed after 24 and 48 h, while those for SO-Treatment 2 and DT-Treatment 3 were only analyzed when the complete inactivation of the larvae occurred in the respective treatments. A total of 120 (40 for each replicate) fillets in MA-Treatment 1, 60 (20 for each replicate) fillets in SO-Treatment 2, and 30 (10 for each replicate) fillets in DT-Treatment 3 were tested.

The consumer acceptance of the marinated anchovy fillets with the 3 different treatments was established by sensory analysis inspired by Ksouda et al. [54]. Marinated anchovy fillets were served at room temperature (20 ± 1 °C), under normal light conditions in white porcelain trays coded with random digit numbers. A panel of fifteen untrained, random people of both genders selected among the staff and students of the Department of Veterinary Sciences, University of Messina (Italy), evaluated the influence of LMN on the typical “odor”, “color”, and “taste” of the marinated anchovy fillets using the following demerit scoring: 0 for “typical”; 1 for “just perceptible”; 2 for “moderate”; 3 for “intense”. All respondents have consented to the participation in the survey. For each treatment, marinated anchovy fillets treated without LMN (0%) were used as control. It is important to stress that the sensory evaluation conducted in this study was based on a hedonistic analysis that requires a much greater number of people than those herein involved in order to perform a solid and representative statistical evaluation of the results obtained. Therefore, the present evaluation has had only an exploratory purpose, and more in-depth investigations are necessary to understand the real influence of the LMN on the sensory profile of the marinated anchovy fillets.

### 2.9. Data Processing

For each treatment, the means of the viability scores assigned to the larvae were normalized and expressed as a percentage according to the following equation:

Normalized viability score (%) = (Mean viability score × 100)/3 (1)

The normalized viability scores (%) were plotted against time to show the anisakicidal efficacy of each treatment at the various time points. The results of the replicates obtained at each time point for each treatment were expressed as the mean value of the normalized viability scores (%) ± standard deviation (%).

To evaluate any significant differences in the anisakicidal activity of the different LMN concentrations in each treatment, statistical analysis was carried out comparing the mean viability scores of the *Anisakis* larvae at each time point. Ordinary one-way ANOVA was used to assess the anisakicidal efficacy of the different LMN concentrations both in the MA and SO, whereas, for the DT, paired *t*-test was used to assess significant differences between treated and control samples. The normality distribution of data was evaluated by the Kolmogorov–Smirnov test, and Tukey’s honestly significant difference test was used for the multiple comparisons within the obtained ANOVA data. The critical significance level (*p*) was set at 5% (0.05), and all tests were two sided. Statistical analyses were performed using GraphPad Prism version 9.1.1 for Windows (GraphPad Software, San Diego, CA, USA, www.graphpad.com, accessed date: 1 March 2022).

## 3. Results

### 3.1. Anisakis Molecular Identification

The results of the molecular analysis confirmed the morphological identification of all the larvae as *Anisakis* L3 type I. In fact, of the 14 larvae analyzed, 13 were identified as *A. pegreffii* (92.86%) and 1 as a hybrid between *A. pegreffii* and *A. simplex* (Figure S2).

### 3.2. Treatment 1: Addition of LMN during Marinating at 4 °C

**Figure 2:**
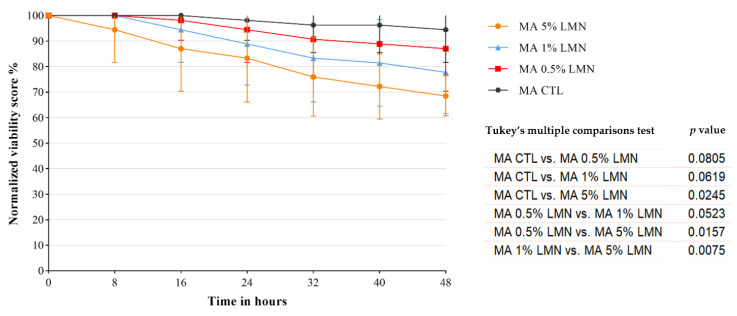
MA-Treatment 1: normalized mean viability score (%) over time of *Anisakis* larvae experimentally parasitized in anchovy fillets treated with different R (+) limonene (LMN) concentrations (5%, 1%, 0.5%) in a typical industrial marinating solution and relative control (CTL) without LMN. The results of the statistical analysis performed for the different treatments are also reported.

No dead *Anisakis* larvae were observed for all the tested LMN concentrations in MA-Treatment 1 until 48 h of exposure but only a reduction of their viability was found (Table S1), which was significantly higher in those exposed to 5% of LMN (*p* < 0.05), while no significant differences were observed between the other LMN concentrations (*p* > 0.05). At the end of the treatment, the normalized mean viability score of each LMN concentration was: 68.52 ± 7.86% at 5%, 77 ± 16.17% at 1%, 87.04 ± 16.72% at 0.5%, and 94.44 ± 12.78% at 0% (MA-CTL) (Figure 2). Under the stereomicroscope, no injuries were observed in all the examined *Anisakis* larvae.

### 3.3. Treatment 2: Addition of LMN during Storage in Sunflower Seed Oil at 4 °C

**Figure 3:**
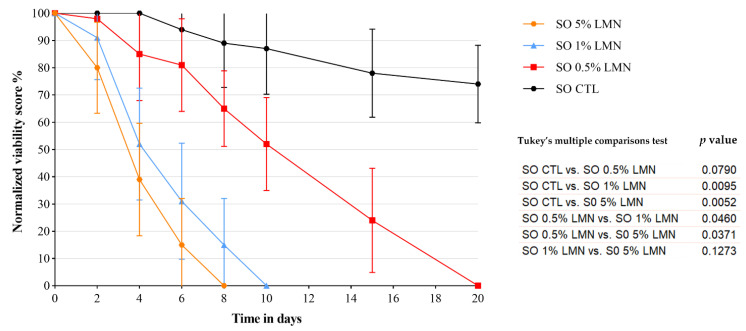
SO-Treatment 2: normalized mean viability score (%) over time of *Anisakis* larvae experimentally parasitized in anchovy fillets treated with different R (+) limonene (LMN) concentrations (5%, 1%, 0.5%) during storage in sunflower seed oil and relative control (CTL) without LMN. The results of the statistical analysis performed for the different treatments are also reported.

All the LMN concentrations tested in SO-Treatment 2 determined a complete devitalization of the *Anisakis* larvae (Figure 3). In particular, the total larvae devitalization occurred after the 8th, 10th, and 20th day of treatment at 5%, 1%, and 0.5% of LMN, respectively (Table S2). In the SO-CTL, until the 20th day, no complete inactivation of the larvae was observed. No significant difference was observed between the anisakicidal effect of 5% and 1% of LMN (*p* > 0.05), but both were significantly more effective than 0.5% LMN (*p* < 0.05).

**Figure 4:**
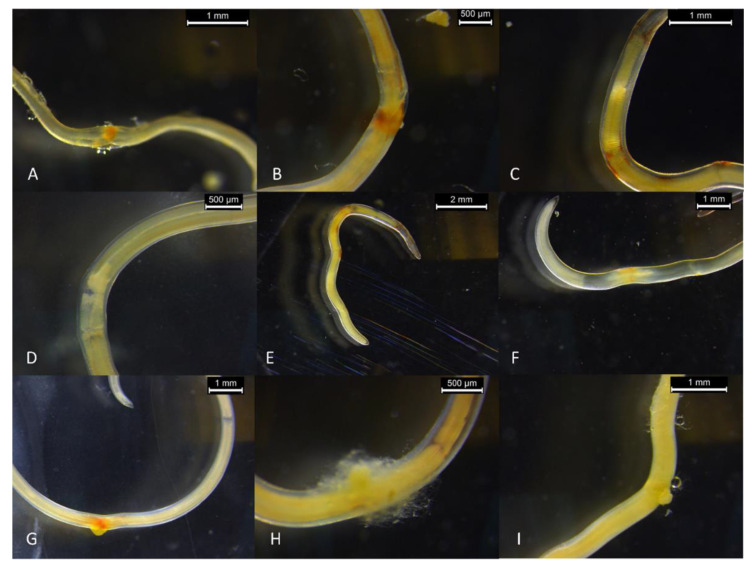
Examples of damages and lesions observed under a stereomicroscope in *Anisakis* larvae experimentally parasitized in anchovy fillets treated with different concentrations of limonene during the traditional industrial marinating process: (**A**) continuous lesions of the cuticle; (**B**) continuous lesions of the cuticle; (**C**) slight damages of the cuticle; (**D**) interruptions of the digestive tract; (**E**) slight damages of the cuticle and interruptions of the digestive tract; (**F**) interruptions of the digestive tract; (**G**) continuous lesions of the cuticle associated with leak of the digestive tract; (**H**) continuous lesions of the cuticle associated with leak of the digestive tract. In picture (**I**), the absence of lesions in *Anisakis* larvae in a control sample.

Under the stereomicroscope, different lesions were observed in the dead *Anisakis* larvae treated with LMN (Figure 4). The number of larvae affected was proportional to the LMN concentration. In decreasing order, at 5% of LMN the alterations observed were as follows: (i) slight damages of the cuticle in the 61% (number of larvae = 77) of the larvae; (ii) interruptions of the digestive tract in the 57% (n = 72) of larvae; (iii) continuous lesions of the cuticle in the 41% (n = 52) of larvae; (iv) continuous lesions of the cuticle associated with a leak of the digestive tract in the 22% (n = 28) of larvae. In increasing order, at 1% of LMN, the alterations observed were as follows: (i) slight damages of the cuticle in the 40% of larvae (n = 50); (ii) interruptions of the digestive tract in the 33% of larvae (n = 42); (iii) continuous lesions of the cuticle in the 15% of larvae (n = 19); (iv) continuous lesions of the cuticle associated with a leak of the digestive tract in the 5% of larvae (n = 6). Finally, at 0.5% of LMN, the inactivated parasites showed only slight damages of the cuticle (25% of larvae; n = 31) and interruption of the digestive tract (15% of larvae; n = 19).

### 3.4. Treatment 3: Double Treatment with LMN

The LMN concentration at 1% was selected and tested in the DT-Treatment 3 considering the anisakicidal efficacy shown in SO-Treatment 3 and the lower influence on the organoleptic properties of the anchovy fillets.

**Figure 5:**
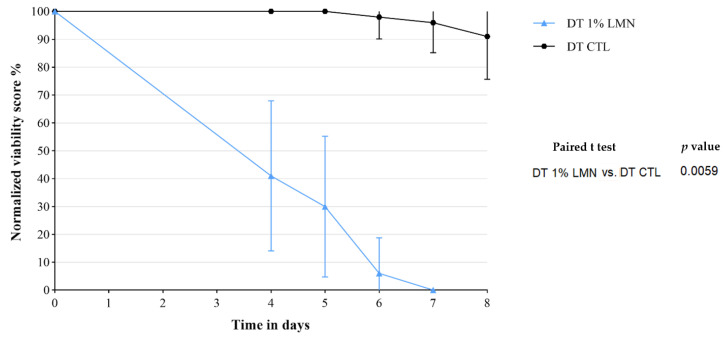
DT-Treatment 3: normalized mean viability score (%) over time of *Anisakis* larvae experimentally parasitized in anchovy fillets exposed to R (+) limonene (LMN) at 1% in a double treatment (DT) both during the marinating process and subsequent storage in sunflower seed oil. The results of the statistical analysis performed for the different treatments are also reported.

The complete devitalization of the larvae occurred just after the 7th day of treatment (Table S3). Even after 6 days, the viability of the larvae was substantially compromised with a relative normalized mean score of 5.56 ± 12.78% (Figure 5). Under stereomicroscope, the devitalized *Anisakis* larvae showed, in decreasing order: (i) slight damages of the cuticle in the 48% of larvae (n = 53); (ii) interruptions of the digestive tract in the 43% of larvae (n = 39); (iii) continuous lesions of the cuticle in the 24% of larvae (n = 22); (iv) continuous lesions of the cuticle associated with a leak of the digestive tract in the 8% of larvae (n = 7).

### 3.5. Sensory Analysis

**Figure 6:**
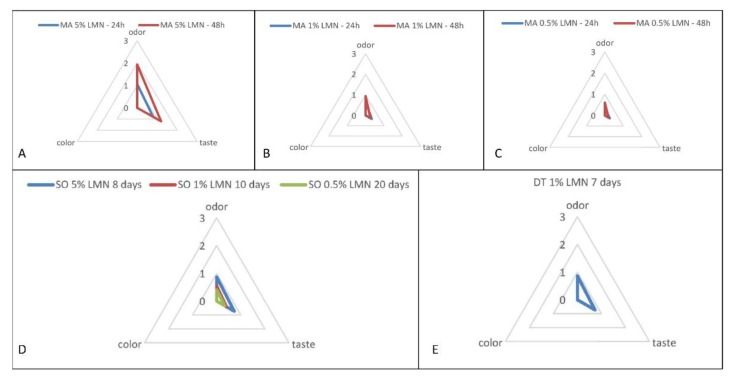
Sensory analysis (odor, color, and taste) of anchovy fillets treated with several R (+) limonene (LMN) concentrations in three different treatments: (**A**) 5% of LMN in marinating solution a 4 °C (MA-Treatment 1); (**B**) 1% of LMN in marinating solution a 4 °C (MA-Treatment 1); (**C**) 0.5% of LMN in marinating solution a 4 °C (MA-Treatment 1); (**D**) 5%, 1%, and 0.5% of LMN in sunflower seed oil (SO-Treatment 2); (**E**) 1% of LMN a double treatment both during marinating process as and subsequent storage in sunflower seed oil (DT-Treatment 3). This survey was carried out by a panel of fifteen untrained, random people of both genders.

The results obtained from the sensory analysis are shown in Figure 6. Overall, nonrelevant variations of the typical color were described by the panelists in fillets treated with LMN compared to the control ones, for all treatments and concentrations.

The influence of 5% LMN on the odor and taste of the fillets was greater than the that of other LMN concentrations both in MA-Treatment 1 and SO-Treatment 2. No differences were observed between odor and taste scores of the fillets treated with 1% and 0.5% LMN and control, both in MA-Treatment 1 and SO-Treatment 2 as well as between 1% LMN and control samples in DT-Treatment 3. Panelists reported that fillets treated with 5% of LMN in MA-Treatment 1 had a just perceptible odor and taste of lemon after 24 h of exposure, whereas, after 48 h, the lemon odor had become moderate, and the lemon taste had slightly increased. Regarding the other LMN concentrations tested in MA-Treatment 1, after 24 h, the odor and taste were essentially typical for 1% as well as for 0.5% even after 48 h, while a just perceptible lemon odor without significant taste change was appreciated for 1% after 48 h.

In SO-Treatment 2, a just perceptible odor and taste of lemon were appreciated by panelists on the 8th day in fillets treated with 5% of LMN, whereas no sensory variations were perceived on the 10th and 20th of treatment in fillets exposed to 1% and 0.5% of LMN, respectively.

Finally, the addition of 1% LMN in the DT-Treatment 3 did not substantially change the sensory properties of the fillets compared to the control ones on the 7th day of treatment. The odor and taste in all the treated fillets, even those exposed to the highest concentration (5%), were appreciated by all the panelists.

## 4. Discussion

Although only a few larvae were analyzed, biomolecular analysis, confirmed the prevalence of *A. pegreffii* and the presence of *A. pegreffii* and *A. simplex* hybrid forms in the *L. caudatus* fished in the Mediterranean Sea [45].

The results of the present study show how LMN used in the preparation of marinated anchovy fillets is able to devitalize *Anisakis* larvae experimentally parasitized in the flesh of the fillets without compromising, potentially, their sensory characteristics.

LMN is a monocyclic terpene produced by more than 300 plants across the world. It plays a key role in plant defense against insects and pathogens, and it is involved in several hormonal signals [43]. Among terpenes, LMN represents the major component of most oils and essential oils obtained from oranges, grapefruits, and lemons, and there is ample evidence of its antioxidant, antimicrobial, antifungal, anti-inflammatory, anticarcinogenic, and nematicidal activities [55,56]. Nowadays, LMN is widely used as a flavoring agent in perfumes, creams, soaps, and household cleaning products, and, according to Regulation (EC) No. 872/2012 [57], it can be used in food as a flavor additive without any restrictions. Against this background, the bactericidal efficiency of LMN was explored against pathogens and specific spoilage organisms in different foods [58,59,60,61]. With reference to seafood, LMN was used in a mixture with sunflower seed oil to evaluate its effects on the spoilage flora of fresh sea bream (*Sparus aurata*) fillets stored under vacuum and refrigeration [61]. Shelf life of the sea bream fillets was extended to approximately 6–9 days, and their taste and odor resembled that of lemon as also described in the present study by the panelists for the treated anchovy fillets. Indeed, the fragrance of LMN may not be a limit in those foods that are usually seasoned with lemon such as marinated anchovy fillets, which are usually flavored with lemon in homemade preparations or with citric acid at the industrial level.

The results obtained in our previous in vitro study have already revealed a significant activity of LMN against *Anisakis* larvae when added to a typical industrial marinating solution [34]. In this regard, total *Anisakis* larvae inactivation was observed after 16 h at 5% and 24 h at 1% and 0.5% of LMN. Although a steady inactivation trend was also observed in sunflower seed oil, the addition of different concentrations of LMN did not completely inactivate the larvae even after 7 days of treatment in vitro. Interestingly, the results of the present study are not only different but substantially opposite to those observed in vitro. In the present study, the addition of LMN in the marinating solution (MA-Treatment 1) during the anchovy fillet marinating process did not reveal an anisakicidal effect but only a vitality reduction at each LMN concentration tested. In fact, all the experimentally encysted larvae were still alive after 2 days of treatment. On the other hand, the use of 5%, 1%, and 0.5% of LMN in sunflower seed oil (SO-Treatment 2) resulted in the 100% inactivation of the *Anisakis* larvae within 8, 10, and 20 days of treatment, respectively. An even higher anisakicidal effect was observed for 1% LMN when used in the DT-Treatment 3 with the total larvae inactivation achieved just after 1 week.

The lack of LMN efficiency during the marinating process (MA-Treatment 1) compared to the in vitro test may be related to a reduction in the compound diffusion within the fillets, which acts as a protection for the larvae. It is also important to consider the stability of LMN in the media tested. It is known that, under acid conditions, such as those of the marinating solution, LMN can degrade into other terpenes such as α-terpineol; trans and cis 1,8 terpinene; 1,4 or 1,8 cineole; etc., which may be responsible for the anisakicidal activity observed in vitro [43]. Conversely, these compounds may not be able to diffuse into fish tissues and reach the *Anisakis* larvae. There are no studies on the stability of LMN in oil; therefore, we could speculate that either it spreads as it is in the fish tissues or it is degraded into compounds that still manage to reach the larvae present in the flesh of the anchovy.

As with other biological properties, the anisakicidal activity of LMN could be related to different mechanisms of action depending on the medium tested, the treatments it undergoes, and the metabolites that could be formed.

On one hand, these differences might also explain the variability in the type and frequency of lesions observed between treatments. On the other hand, the lesions observed were similar in each treatment suggesting that, regardless of the compound involved, the effects on the larvae were similar. Indeed, similar lesions were observed in other in vitro treatments of the *Anisakis* larvae with flavored olive oils aromatized with rosemary, cinnamon, cardamon, and laurel [38], essential oils of *Tagetes minuta* [35], *Matricaria chamomilla* [62], *Thymus vulgaris* [33], and *Nepeta cataria* [36].

Understanding the stability of LMN during food production and storage is also crucial to establishing the toxicological effects and risks associated with its use. Although LMN has been generally recognized as a safe substance [57,63], some secondary degradation compounds may have high toxic effects on humans [43]. For example, the use of ozone in fruit juice processing degrades LMN, present in high quantities in these foodstuffs, into 3-isopropenyl-6-oxoheptanal, 4-acetyl-1-methylcyclohexane, and 4-oxopentanal, which are known to be toxic to lung cells [64]. There is substantial evidence that many other metabolites can be formed from LMN after food processing, but information about their toxicity is not available. Furthermore, LMN also shows few toxic effects, especially hepatotoxicity and neurotoxicity, even if more relevant and detailed studies are needed to better characterize these effects [43].

Finally, it should be also considered that all known treatments (including freezing) capable of devitalizing *Anisakis* larvae are not able to resolve the risk of allergic response. In fact, the exposure to nematode materials remaining in the treated product can still determine allergic-type immune responses without infection via viable larvae [65]. In this regard, Speciale et al. [14] have shown how crude extracts of devitalized (even by using biocides) larvae induce a strong inflammation response in intestinal epithelial cells also exacerbating the effects of other inflammatory stimuli.

## 5. Conclusions

The present study has highlighted the anisakicidal activity of LMN, which is able to devitalize *Anisakis* larva experimentally parasitized in the flesh of marinated anchovy fillets. The taste and aroma of lemon are the main organoleptic modifications in the treated fillets, which were nevertheless appreciated by the fifteen panelists. The sensory qualities potentially compatible with use in fishery products and the observed anisakicidal efficacy make LMN a possible natural alternative to the freezing treatments required by current regulations for the consumption of raw or practically raw fish products. However, further studies are needed to understand the stability of LMN during the processing and storage of marinated anchovy fillets and the toxicity arising from its degradation products.

## Figures and Tables

**Table 1:** Evaluation of the *Anisakis* “viability score” inspired by the classification of Hirasa and Takemasa [51] implemented according to Guan et al. [50].

Viability Score	Score Descriptor	Criteria
3	Viable	In situ movement of the whole larval body
2	Reduction of motility	In situ movement of at least one part of the larval body
1	Motile only after stimulation	In situ movement of at least one part of the larval body only after mechanical stimulation
0	Death	No in situ movement

## Data Availability

Data is contained within the article or Supplementary Materials.

## Supplementary Materials

The following supporting information can be downloaded at https://www.mdpi.com/article/10.3390/foods11081121/s1: Figure S1: Flow chart of the traditional industrial marinating process of anchovy fillets; Figure S2: Agarose gel electrophoresis of the amplification products of *Anisakis* spp. specimens using the restriction patterns *HinfI* and *HhaI*; Table S1: Viability scores over time of *Anisakis* larvae exposed to MA-Treatment 1; Table S2: Viability scores over time of *Anisakis* larvae exposed to SO-Treatment 2; Table S3: Viability scores over time of *Anisakis* larvae exposed to DT-Treatment 3.
