# Supplementary material for: Anisakicidal Effects of R (+) Limonene: An Alternative to Freezing Treatment in the Industrial Anchovy Marinating Process
Source: Foods. 2022 Apr 13;11(8):1121. doi: 10.3390/foods11081121 (PMC9028723; doi:10.3390/foods11081121)
Supplement: Supplementary file 1 [file foods-11-01121-s001.zip › Figures S1 and S2.pdf]

Supplementary Files

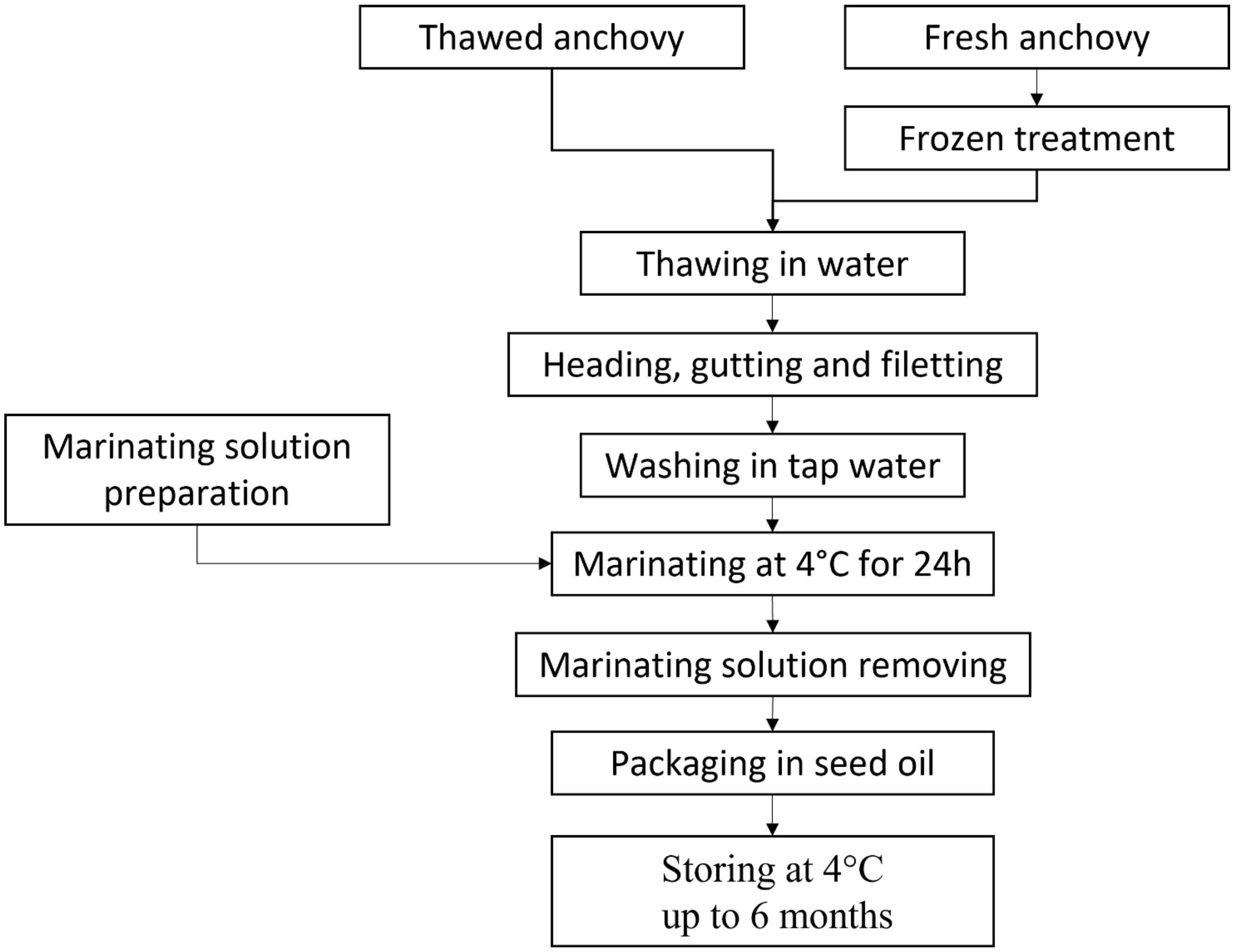

Figure S1. Flow chart of the traditional industrial marinating process of anchovy fillets.

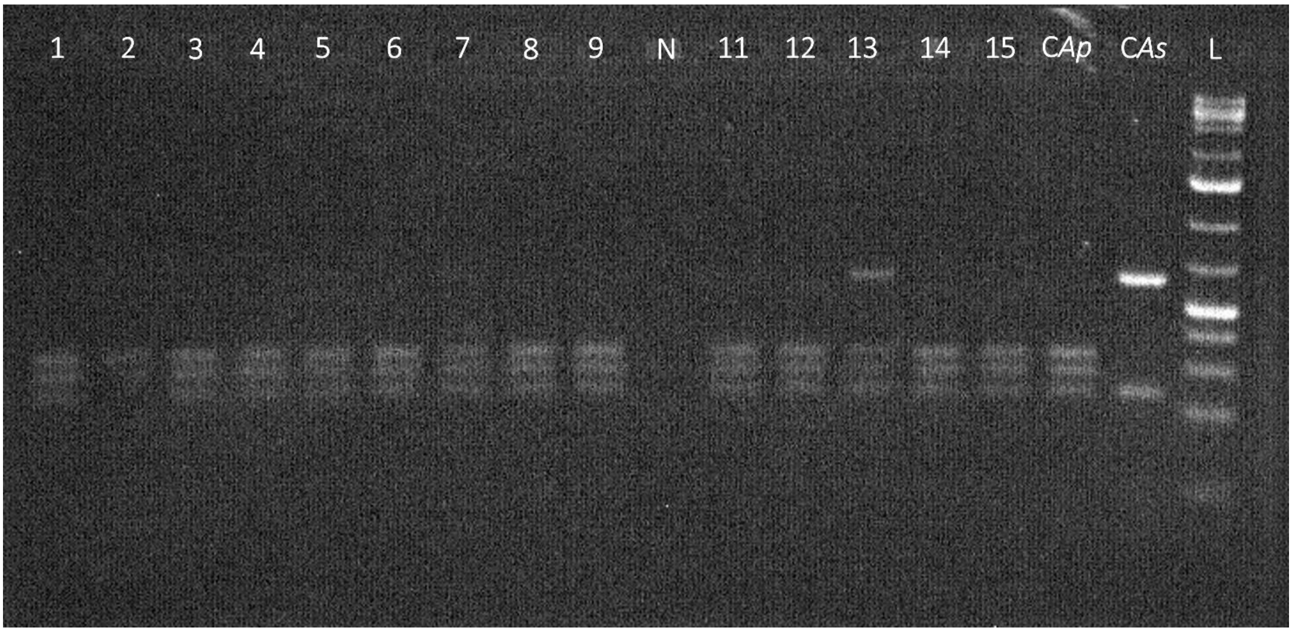

Figure S2. Agarose gel electrophoresis of the amplification products of *Anisakis* spp. specimens using the restriction patterns *HinfI* and *HhaI*. Lanes 1-9: *A. pegreffii*, Lane 10: negative control, Lanes 11-12: *A. pegreffii*, Lane 13: *A. pegreffi* × *A. simplex* s.s. hybrid, Lanes 14-15: *A. pegreffii*, Lane 16: *A. pegreffii* positive control, Lane 17: *A. simplex* s.s. positive control, L: ladder.
