# Supplementary material for: Anisakicidal Effects of R (+) Limonene: An Alternative to Freezing Treatment in the Industrial Anchovy Marinating Process
Source: Foods. 2022 Apr 13;11(8):1121. doi: 10.3390/foods11081121 (PMC9028723; doi:10.3390/foods11081121)
Supplement: Supplementary file 1 [file foods-11-01121-s001.zip › Table S1.pdf]

Table S1. Viability scores over time of Anisakis larvae exposed to MA-Treatment 1 Number of specimens and relative viability score over time of *Anisakis* larvae experimentally parasitized in anchovy fillets treated with R (+) Limonene during marinating at 4°C (MA-Treatment 1).

**LMN**	**Viability score**	**8 h**			**16 h**			**24 h**			**32 h**			**40 h**			**48 h**			**Total n. *Anisakis* (n. fillets)**
		**r.1**	**r.2**	**r.3**	**r.1**	**r.2**	**r.3**	**r.1**	**r.2**	**r.3**	**r.1**	**r.2**	**r.3**	**r.1**	**r.2**	**r.3**	**r.1**	**r.2**	**r.3**	
**5%**	**3**	5	6	4	4	4	3	3	3	3	2	1	2	1	1	1	1	-	-	
	**2**	1	-	2	2	2	3	3	3	3	4	5	4	5	5	5	5	6	6	n. 108
	**1**	-	-	-	-	-	-	-	-	-	-	-	-	-	-	-	-	-	-	(n. 54)
	**0**	-	-	-	-	-	-	-	-	-	-	-	-	-	-	-	-	-	-	
**1%**	**3**	6	6	6	5	5	5	4	4	4	4	2	2	4	2	2	2	2	2	
	**2**	-	-	-	1	1	1	2	2	2	2	4	4	2	4	4	4	4	4	108
	**1**	-	-	-	-	-	-	-	-	-	-	-	-	-	-	-	-	-	-	(n. 54)
	**0**	-	-	-	-	-	-	-	-	-	-	-	-	-	-	-	-	-	-	
**0.5%**	**3**	6	6	6	6	6	5	5	5	5	5	4	4	5	4	3	4	3	4	
	**2**	-	-	-	-	-	1	1	1	1	1	2	2	1	2	3	2	3	2	108
	**1**	-	-	-	-	-	-	-	-	-	-	-	-	-	-	-	-	-	-	(n. 54)
	**0**	-	-	-	-	-	-	-	-	-	-	-	-	-	-	-	-	-	-	
**Control**	**3**	6	6	6	6	6	6	5	6	6	5	5	6	5	5	6	5	5	5	
	**2**	-	-	-	-	-	-	1	-	-	1	1	-	1	1	-	1	1	1	108
	**1**	-	-	-	-	-	-	-	-	-	-	-	-	-	-	-	-	-	-	(n. 54)
	**0**	-	-	-	-	-	-	-	-	-	-	-	-	-	-	-	-	-	-	
																			**Total number of Anisakis**	**432**
																			**Total number of anchovy fillets**	**216**

r.= replication.
LMN= Limonene
For each concentration and replication n. 36 *Anisakis* larvae experimentally parasitized in 18 anchovy fillets.
