# Supplementary material for: Anisakicidal Effects of R (+) Limonene: An Alternative to Freezing Treatment in the Industrial Anchovy Marinating Process
Source: Foods. 2022 Apr 13;11(8):1121. doi: 10.3390/foods11081121 (PMC9028723; doi:10.3390/foods11081121)
Supplement: Supplementary file 1 [file foods-11-01121-s001.zip › Table S2.pdf]

Table S2. Viability scores over time of Anisakis larvae exposed to SO-Treatment 2 Number of individuals and relative viability score over time of *Anisakis* larvae experimentally parasitized in anchovy fillets treated with R (+) Limonene during storage in sunflower seed oil at 4°C (SO–Treatment 2).

**LMN**	**Viability score**	**2nd day**			**4th day**			**6th day**			**8th day**			**10th day**			**15th day**			**20th day**			**Total n. *Anisakis* (n. fillets)**
		**r.1**	**r.2**	**r.3**	**r.1**	**r.2**	**r.3**	**r.1**	**r.2**	**r.3**	**r.1**	**r.2**	**r.3**	**r.1**	**r.2**	**r.3**	**r.1**	**r.2**	**r.3**	**r.1**	**r.2**	**r.3**	
	**3**	3	2	2	-	-	-	-	-	-	-	-	-	-	-	-	-	-	-	-	-	-	
**5%**	**2**	3	4	4	2	1	2	-	-	-	-	-	-	-	-	-	-	-	-	-	-	-	126
	**1**	-	-	-	4	4	3	3	2	3	-	-	-	-	-	-	-	-	-	-	-	-	(n. 63)
	**0**	-	-	-	-	1	1	3	4	3	**24**	**24**	**24**	-	-	-	-	-	-	-	-	-	
	**3**	5	4	4	1	-	-	-	-	-	-	-	-	-	-	-	-	-	-	-	-	-	
**1%**	**2**	1	2	2	2	3	3	1	1	1	-	-	-	-	-	-	-	-	-	-	-	-	126
	**1**	-	-	-	3	3	3	4	3	4	3	2	3	-	-	-	-	-	-	-	-	-	(n. 63)
	**0**	-	-	-	-	-	-	1	2	1	3	4	3	**18**	**18**	**18**	-	-	-	-	-	-	
	**3**	6	5	6	4	3	3	3	2	3	-	1	-	-	-	-	-	-	-	-	-	-	
**0.5%**	**2**		1	-	2	3	3	3	4	3	5	5	5	4	3	3	1	-	-	-	-	-	126
	**1**	-	-	-	-	-	-	-	-	-	1	-	1	2	3	3	3	3	5	-	-	-	(n. 63)
	**0**	-	-		-	-	-	-	-	-	-	-	-	-	-	-	2	3	1	**6**	**6**	**6**	
	**3**	6	6	6	6	6	6	5	6	4	4	4	4	3	4	4	3	2	1	1	2	1	
**Control**	**2**	-	-	-	-	-	-	1	-	2	2	2	2	3	2	2	3	4	5	5	4	5	126
	**1**	-	-	-	-	-	-	-	-	-	-	-	-	-	-	-	-	-	-	-	-	-	(n. 63)
	**0**	-	-	-	-	-	-	-	-	-	-	-	-	-	-	-	-	-	-	-	-	-	
**Total number of Anisakis**																							**504**
**Total number of anchovy fillets**																							**252**

r.= replication.
LMN= Limonene
For each concentration and replication n. 42 *Anisakis* larvae experimentally parasitized in 21 anchovy fillets.
