# Supplementary material for: Anisakicidal Effects of R (+) Limonene: An Alternative to Freezing Treatment in the Industrial Anchovy Marinating Process
Source: Foods. 2022 Apr 13;11(8):1121. doi: 10.3390/foods11081121 (PMC9028723; doi:10.3390/foods11081121)
Supplement: Supplementary file 1 [file foods-11-01121-s001.zip › Table S3.pdf]

Table S3. Viability scores over time of Anisakis larvae exposed to DT-Treatment 3 Number of individuals and relative viability score over time of *Anisakis* larvae experimentally parasitized in anchovy fillets exposed to R (+) Limonene at 1% in a double treatment (DT – Treatment 3) both during the marinating process and subsequent storage in sunflower seed oil.

**LMN in DT**	**Viability score**	**4th day**			**5th day**			**6th day**			**7th day**			**8th day**			**Total n. *Anisakis* (n. fillets)**
		**r.1**	**r.2**	**r.3**	**r.1**	**r.2**	**r.3**	**r.1**	**r.2**	**r.3**	**r.1**	**r.2**	**r.3**	**r.1**	**r.2**	**r.3**	
**1%**	3	-	-	-	-	-	-	-	-	-	-	-	-	-	-	-	90 (n. 45)
	2	4	2	1	2	1	1	-	-	-	-	-	-	-	-	-	
	1	1	2	3	2	3	3	1	1	1	-	-	-	-	-	-	
	0	1	2	2	2	2	2	5	5	5	**14**	**14**	**14**	-	-	-	
**Control**	3	6	6	6	6	6	6	6	6	5	5	6	5	4	5	4	90 (n. 45)
	2	-	-	-	-	-	-	-	-	1	1	-	1	2	1	2	
	1	-	-	-	-	-	-	-	-	-	-	-	-	-	-	-	
	0	-	-	-	-	-	-	-	-	-	-	-	-	-	-	-	
																**Total number of Anisakis**	**180**
																**Total number of anchovy fillets**	**90**

r.= replication.
LMN=Limonene.
For each concentration and replication n. 30 *Anisakis* larvae experimentally parasitized in 15 anchovy fillets.
